# Tumeur bénigne de la cavité buccale: granulome idiopathique de la langue

**DOI:** 10.11604/pamj.2018.31.199.16165

**Published:** 2018-11-22

**Authors:** Ines Kechaou, Imène Boukhris

**Affiliations:** 1 Service de Médecine Interne B, Hôpital Charles Nicolle, Université de Tunis El Manar, Faculté de Médecine de Tunis, Tunisie

**Keywords:** Granulome, langue, tumeur bénigne, Granuloma, tongue, benign tumor

## Abstract

The diagnosis of granuloma of the oral cavity can favour the detection of several diseases such as sarcoidosis, Crohn's disease, tuberculosis, syphilis and malignant tumors. In Melkerson Rosenthal syndrome it has an idiopathic origin. In this regard, we here report an original case of idiopathic granuloma of the tongue not secondary to Melkerson Rosenthal syndrome and being part of benign tumors of the oral cavity. The study involved a 60-year old female patient, with no particular past medical history, presenting with discomfort during mastication secondary to a swelling on the free edge of the tongue which had occurred one month before her admission. Physical examination showed overgrowth at the level of the right edge of the tongue measuring 15 mm along its longer axis. The tongue was not plicated and there was no cervical adenopathy. The remainder of the physical examination was normal. MRI of the oral cavity showed suspected necrotizing tissue lesion on the right free edge of the mobile tongue measuring 15x12x19 mm without signs of extension to the ipsilateral lingual pedicle or to the floor of the oral cavity and without cervical adenomegalies. Biopsy of tongue lesion showed gigantocellular epithelioid granulomas without caseous necrosis or histological signs of malignancy. Biologically, there was no inflammatory syndrome, leukopenia or lymphopenia. The assessment of an underlying cause was negative: syphilis serology, evaluation of phosphocalcic profile, dosing of the angiotensin-converting enzyme, intradermo tuberculin reaction, chest radiograph, thoracoabdominopelvic CT scan and ophthalmological examination. Finally, the diagnosis of idiopathic granuloma was retained in the absence of arguments in favor of its secondary nature.

## Image en médecine

Le granulome de la cavité buccale constitue la circonstance de découverte de plusieurs pathologies comme la sarcoïdose, la maladie de Crohn, la tuberculose, la syphilis et les tumeurs malignes. Son origine idiopathique est classique dans le syndrome de Melkerson Rosenthal. A ce propos, nous rapportons l'observation originale d'un granulome idiopathique de la langue non secondaire à un syndrome de Melkerson Rosenthal et faisant partie des tumeurs bénignes de la cavité buccale. Il s'agit d'une patiente âgée de 60 ans sans antécédents pathologiques particuliers qui a présenté un mois avant son admission une gêne au cours de la mastication secondaire à une tuméfaction au niveau du bord libre de la langue. A l'examen, elle avait une excroissance au niveau du bord latéral droit de la langue faisant 15mm de grand axe. Sa langue n'était pas plicaturée et il n'y avait pas d'adénopathies cervicales. Le reste de l'examen somatique était sans particularités. L'IRM de la cavité buccale avait montré une lésion tissulaire nécrosée du bord libre droit de la langue mobile, d'allure suspecte mesurant 15x12x19mm sans signes d'extension au pédicule lingual homolatéral ni au plancher buccal et sans adénomégalies cervicale. La biopsie de la lésion de la langue avait montré des granulomes épithélioïdes et giganto-cellulaires sans nécrose caséeuse et sans signes histologiques de malignité. Sur le plan biologique, il n'y avait pas de syndrome inflammatoire, ni de leucopénie ou de lymphopénie. Le bilan étiologique à la recherche d'une étiologie sous-jacente était négatif: sérologie syphilis, bilan phosphocalcique, dosage de l'enzyme de conversion de l'angiotensine, intradermo réaction à la tuberculine, radiographie du thorax, scanner thoraco-abdomino pelvien et examen ophtalmologique. Finalement, le diagnostic de granulome idiopathique a été retenu devant l'absence d'arguments en faveur de son caractère secondaire.

**Figure 1:**
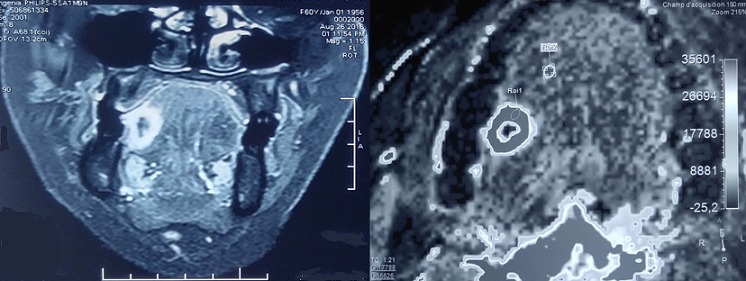
IRM montrant une lésion tissulaire du bord libre de la langue mobile: cette lésion est en hypersignal T2 intermédiaire avec un centre nécrosé

